# Serum profiling of uPA, PAI-1, and suPAR in systemic sclerosis: a preliminary study on analytical aspects and associations with microvascular and fibrotic manifestations

**DOI:** 10.3389/fimmu.2025.1697785

**Published:** 2025-12-02

**Authors:** Filomena Napolitano, Ilaria Mormile, Gabriele Mignogna, Amato de Paulis, Francesca Wanda Rossi, Nunzia Montuori

**Affiliations:** 1 Department of Translational Medical Sciences, University of Naples Federico II, Naples, Italy; 2 Center for Basic and Clinical Immunology Research (CISI), World Allergy Organization (WAO) Center of Excellence, University of Naples Federico II, Naples, Italy

**Keywords:** urokinase, plasminogen activator inhibitor-1, soluble urokinase plasminogen activator, systemic sclerosis, fibrosis, microvascular abnormalities

## Abstract

**Introduction:**

In systemic sclerosis (SSc), the laboratory panel lacks biomarkers able to predict the disease course and/or reflect the fibrotic activity in the skin and internal organs. We assessed the association of the serum levels of urokinase (urokinase plasminogen activator, uPA), plasminogen activator inhibitor-1 (PAI-1), and soluble urokinase plasminogen activator receptor (suPAR) with the microvascular and fibrotic manifestations.

**Methods:**

A total of 21 patients with SSc were enrolled in the study. The serum levels of PAI-1, uPA, and suPAR were measured using ELISA, and the diagnostic performance of two widely debated suPAR ELISA kits, i.e., Human suPAR ELISA (Biovendor R&D, Brno, Czech Republic) and suPARnostic ELISA (Virogates, Copenaghen, Denmark), was examined in patients with SSc and in healthy controls. The serum uPA, PAI-1, and suPAR levels were correlated with the typical fibrotic and vascular markers, such as TGF-β1 and VEGF-A, the anti-Scl-70 antibodies, and the nailfold capillaroscopic abnormalities and pulmonary function indicators.

**Results:**

Lower circulating uPA (3,226 ± 2,444 pg/ml) and higher PAI-1 (95.30 ± 22.80 ng/ml) and suPAR (2.522 ± 1.186 ng/ml with Human suPAR ELISA and 3.835 ± 2.944 ng/ml with suPARnostic ELISA) levels were found in patients with SSc. Both suPAR assays showed high sensitivity and specificity for SSc. However, suPAR measured using the suPARnostic assay displayed stronger associations with the clinical manifestations, indicating its potential as a marker of disease severity. uPA was negatively correlated with TGF-β1 (*p*=0.008), whereas PAI-1 and suPAR (measured with the suPARnostic assay) were positively correlated with VEGF-A (*p*=0.01 and *p*=0.03, respectively). Furthermore, higher suPAR levels obtained with the suPARnostic assay, but not with Human suPAR ELISA, were associated with microvascular and fibrotic manifestations.

**Conclusion:**

This preliminary study provides meaningful evidence supporting the potential of suPAR as a marker of disease severity in SSc and the impact of method-related differences in the levels of suPAR.

## 1 Introduction

Systemic sclerosis (SSc) is a rare rheumatic disease characterized by microvascular injury, autoimmunity, and fibrosis of the skin and internal organs (1, 2). The traditional classification into limited cutaneous (lcSSc) and diffuse cutaneous SSc (dcSSc) may not allow for the stratification of all SSc patients due to the wide disease heterogeneity in these patients (3). The manifestations of SSc significantly reduce the quality of life of affected patients and can be lethal. In particular, interstitial lung disease (ILD), pulmonary artery hypertension, scleroderma–renal crisis, and cardiac involvement are the main causes of SSc-related deaths (4).

The 2013 European League Against Rheumatism (EULAR) and the American College of Rheumatology (ACR) classification criteria are the point of reference for the diagnosis of SSc, although not all patients fulfill them (5).

Recently, the relationship between SSc pathogenesis and the urokinase-type plasminogen activator (uPA) system has been reported (6–10). The uPA system, also known as the fibrinolytic system, plays a key role in the breakdown of the extracellular matrix (ECM) and orchestrates versatile intracellular signaling in a non-proteolytic manner (11). The uPA system consists of a series of activators and inhibitors that primarily regulate the conversion of plasminogen to plasmin. The main components are uPA, plasminogen activator inhibitors 1 and 2 (PAI-1 and PAI-2), and uPA receptor (uPAR) (12). uPA is a serine protease that catalyzes the activation of plasminogen into plasmin, thus regulating ECM remodeling in several physiopathological processes. When uPA binds to uPAR, its activity is localized on the cell surface. uPAR is a highly glycosylated receptor with three homologous domains (from N-terminus DI, DII, and DIII) anchored to the cell surface via the C-terminal glycosylated phosphatidylinositol (GPI). uPAR also interacts with various partners, including vitronectin, integrins, epidermal growth factor receptor (EGFR), platelet-derived growth factor receptor (PDGFR), and G-protein-coupled receptors (GPCRs). uPAR can be cleaved within the DI–DII connecting region, generating the DII–DIII forms of uPAR without ligand-binding properties, except for the formyl peptide receptors (FPRs) (12–14). Both full-length and cleaved DII–DIII forms can be shed from the cell surface and released in a soluble form (i.e., suPAR) in different biological fluids. Elevated suPAR levels in biological fluids have been observed in multiple pathological conditions, including SSc (15–17). PAI-1 is the main suppressor of the catalytic activity of uPA. It reacts both with free and uPAR-associated uPA, thus determining uPA/uPAR endocytosis. After endocytosis, the complex is degraded and only the free form of uPAR is partially recycled to the cell surface (18).

In addition to its physiological role in fibrinolysis through the resolution of fibrin and thrombi, the uPA system is involved in the development of fibrotic disorders (8, 9, 19–21). Accordingly, the aim of this preliminary study was to evaluate the serum levels of uPA, PAI-1, and suPAR and determine their clinical correlations with the fibrotic and vascular complications that occur in patients with SSc.

## 2 Materials and methods

### 2.1 Clinical assessment

In this cross-sectional, single-center cohort study, we evaluated 21 patients with SSc followed at the Division of Internal Medicine and Clinical Immunology of the University of Naples Federico II, Naples, Italy.

The inclusion criteria were age ≥18 years, SSc diagnosis according to the EULAR/ACR 2013 criteria (22), and signature in the written informed consent form. The exclusion criteria were age <18 years, pregnancy, breastfeeding, other systemic autoimmune rheumatic diseases, and known pulmonary and extrapulmonary conditions to minimize their influence on the pulmonary function tests.

We enrolled 10 healthy age- and gender-matched Caucasian subjects as controls with no history of autoimmune or inflammatory diseases. Healthy control participants were recruited voluntarily.

We collected clinical and laboratory data from March 2024 to March 2025. The demographic and clinical data were retrieved from patient medical charts and diaries. The clinical items in the analysis included age, gender, smoking habit, year of diagnosis, details on the extent of skin thickening (puffy fingers and skin thickening extending proximally or distally to the metacarpophalangeal joints), the presence/absence of digital tip ulcers, fingertip pitting scars, calcinosis cutis, Raynaud’s phenomenon, organ involvement (e.g., lung, gastrointestinal, and/or cardiac involvement), and treatments.

Patients were also classified into lcSSc and dcSSc, according to LeRoy et al. (23). For smoking exposure, nonsmokers were classified as zero pack-years, while low, medium, and high smoking exposure were classified as 1–5, 5–10, and greater than 10 pack-years, respectively.

Laboratory data included antinuclear antibodies (ANAs), scleroderma-related antibodies [any of the anti-centromere, anti-topoisomerase I (anti-SCL 70), or anti-RNA polymerase III], other extractable nuclear antigens (ENA, Ro/SSA, La/SSB, anti-Sm, RNP, anti-Jo1, and anti-histone), and C-reactive protein (CRP).

The study was conducted in accordance with the Declaration of Helsinki and was approved by the Ethics Committee of the University of Naples Federico II (protocol no. 348/2018, approved on March 14, 2018). Before participation in the study, patients signed an informed consent form.

### 2.2 Sample collection and storage

Fasting venous blood samples were collected from patients and controls. Serum samples were collected into a separator tube. After clotting for 2 h at room temperature, the samples were centrifuged at 1,000 × *g* for 20 min. Freshly prepared serum samples were immediately assayed or stored in multiple aliquots at −80°C until analysis to avoid repeated freeze–thaw cycles.

### 2.3 ELISAs

Serum uPA was detected using the Human uPA ELISA kit (ELK Biotechnology, Denver, CO, USA) following the manufacturer’s instructions. The serum levels of PAI-1, activated transforming growth factor beta 1 (TGF-β1), and vascular endothelial growth factor A (VEGF-A) were measured with ELISA kits from Elabscience (Elabscience Bionovation Inc., Houston, TX, USA) following the manufacturer’s specifications. The sensitivity of ELISA was 0.067 ng/ml for uPA, 0.1 ng/ml for PAI-1, 18.75 pg/ml for activated TGF-β1, and 18.75 pg/ml for VEGF-A.

Serum suPAR was detected using the Human suPAR ELISA (Biovendor R&D, Brno, Czech Republic) and the suPARnostic assay (Virogates, Copenhagen, Denmark).

The Human suPAR ELISA is a sandwich ELISA that employs a monoclonal mouse antibody coated on a 96-well microplate. The samples were all measured in duplicates and triplicates. All samples had duplicate coefficients of variants <10%.

The suPARnostic assay is a double monoclonal antibody sandwich assay. Microplates are coated with catching rat monoclonal antibody. The samples were all measured in duplicates and triplicates. All samples had duplicate coefficients of variants <10%.

Absorbance was detected at 450 nm with the BGMR-1000 Microplate Reader (Hangzhou Bio-Gener Technology Co., Ltd., Guangzhou, China) within 30 min of stopping the reaction.

### 2.4 Nailfold videocapillaroscopy

Nailfold videocapillaroscopy (NVC) is a noninvasive diagnostic procedure for the *in vivo* study of the structural characteristics of nail fold small vessels (24, 25).

Examination was performed using the CapillaryScope VideoCap 3.0-D1 (DS Medica, Milan, Italy) by the same single experienced operator in order to minimize operator bias. A global evaluation was performed with a ×200 magnification high-resolution objective (26). Cedar oil was applied at the nail fold to enhance the transparency of the epidermis. Fingers with localized trauma were avoided to minimize false-positive patterns (e.g., hemorrhages and abnormal shapes). Before the NVC, patients remained at rest for at least 15 min at a set temperature of 20–22°C to minimize the influence of environmental factors on the examination outcomes. Both hands and the second to the fifth fingers were examined, excluding the thumbs, as suggested by the majority of scholars due to the poor-quality images usually seen at this level (24, 26). At least four images for each finger were captured (i.e., two lateral and two medial fields). Both quantitative and qualitative capillaroscopic parameters were analyzed. The quantitative parameters were as follows: i) the capillary density (number per millimeter), which is the number of capillaries in a 1-mm length of the distal row of the nail fold; ii) the intercapillary distance (in micrometers), which is the distance between two neighboring capillary loops, measured at the widest intercapillary space in the central capillary region; iii) the apical diameter (in micrometers), which is the distance from one external margin of the capillary loop to another on the apex; iv) the internal diameter (in micrometers), which is the distance between the efferent and the afferent loop measured at the same level; v) the external diameter (in micrometers), which is the width of a capillary at its widest section; and vi) the loop length (in micrometers), which is the distance between the apex of a capillary loop and the point where the capillary is no longer visible (24, 26).

The qualitative parameters analyzed were as follows: i) capillary distribution, i.e., the organization of capillaries, scored as regular (0), comma-like (1), irregular (2), and severely deranged (3); ii) capillary morphology, which is the shape of the capillaries, scored as hairpin-like (0), mainly tortuous (1) (i.e., the afferent and efferent limbs bend but do not cross, once crossing shape, twice crossing shape), mainly ramified (2) (i.e., branching, bushy, or coiled capillaries), and with severe alterations (3) (i.e., meandering capillaries and bizarre capillaries) (24, 26); iii) microhemorrhages, i.e., the absence (0) or presence (1) of extra-capillary brown aggregates of clotted blood (27); and iv) interstitial edema, which was scored as absence (0) or presence (1) of fluid accumulation in the interstitial space (28).

Finally, based on the NVC features, the patients were classified into three scleroderma patterns (i.e., early, active, and late) according to Cutolo et al. (24).

### 2.5 Pulmonary assessment

Data on the pulmonary function tests (PFTs) [i.e., spirometry with forced vital capacity (FVC) and diffusing capacity of the lung for carbon monoxide (DLCO)] to identify obstructive, restrictive, or mixed syndrome and to assess any impairments in the exchanges of alveolar gases (29) and high-resolution computed tomography (HRCT) were retrieved from patients’ medical records. PFTs were reported according to the European Respiratory Society (ERS)/American Thoracic Society (ATS) guidelines (30–32). HRCT was defined as negative when no signs of ILD were evident, as determined by the local radiology evaluation.

### 2.6 Statistical analysis

Statistical analyses were performed using the GraphPad Prism 9 software package for Windows (La Jolla, CA, USA). Data were presented as the mean ± standard deviation, median, and range. Categorical variables were presented as number (*n*) and percentages. Statistical analyses were performed using an unpaired *t*-test or the Mann–Whitney test. Correlations between the tested variables were analyzed using Spearman’s correlation and reported as the correlation coefficient (*r*). For heatmap generation, all comparisons were performed using Spearman’s correlation. Statistically significant differences were accepted when the *p*-value was ≤0.05. To control the false discovery rate (FDR) and minimize the risk of false positives, the *p*-values were adjusted for multiple comparisons using the Benjamini–Hochberg (BH) method. Receiver operating characteristic (ROC) curve analysis was performed with the MedCalc software version 11.5.1 (MedCalc Software, Ostend, Belgium).

## 3 Results

### 3.1 Study cohort and demographics

A total of 21 patients (18 women and 3 men) fulfilled the inclusion criteria and were enrolled in this study. Their clinical and main laboratory features are summarized in **Table 1**.

**Table 1:** Clinical and laboratory features in the systemic sclerosis (SSc) cohort (*n*=21).

Features	Values
Female gender, *n* (%)	18 (85.71)
Caucasian ethnicity, *n* (%)	21 (100)
Age (years), mean ± SD (range)	50.2 ± 16.8 (18–72)
Disease duration (years), mean ± SD (range)	5.14 ± 4.63 (1–15)
Smoker, *n* (%)	6 (28.57)
Non-smoker, *n* (%)	11 (52.38)
Former smoker, *n* (%)	4 (19.04)
Low pack-years (<5), *n* (%)	1 (4.76)
Medium pack-years (5–10), *n* (%)	1 (4.76)
High pack-years (>10), *n* (%)	3 (14.28)
Unknown, *n* (%)	2 (9.52)
CRP (mg/dl), reference range 0–05 mg/dl (mean ± SD)	0.2 (0.3)
ANA positivity	21 (100)
Anti-anti-centromere antibody positivity	7 (33.33)
Anti-anti-topoisomerase I (anti-SCL 70) antibody positivity	8 (38.09)
Anti-anti-RNA polymerase III antibody positivity	0 (0)
Anti-Ro/SSA antibody positivity, *n* (%)	2 (9.52)
Anti-La/SSB antibody positivity, *n* (%)	0 (0)
Anti-anti-Sm antibody positivity, *n* (%)	1 (4.76)
Anti-RNP antibody positivity, *n* (%)	1 (4.76)
Anti-anti-Jo1 antibody positivity, *n* (%)	0 (0)
Anti-histone antibody positivity, *n* (%)	0 (0)
Prednisone ≤20 mg/day, *n* (%)	4 (19.04)
Prednisone ≥20 mg/day, *n* (%)	0 (0)
Hydroxychloroquine 400 mg/day, *n* (%)	3 (14.28)
Mycophenolate mofetil 1,000–2,000 mg/week, *n* (%)	17 (80.95)
Nintedanib 300 mg/day, *n* (%)	1 (4.76)
Amlodipine 10 mg/day, *n* (%)	1 (4.76)
Nifedipine 20 mg/day, *n* (%)	1 (4.76)
Bosentan 250 mg/day, *n* (%)	6 (28.57)
Alprostadil^a^, *n* (%)	2 (9.52)
Pentoxifylline 600 mg/day, *n* (%)	2 (9.52)

*CRP*, C-reactive protein; *ANA*, antinuclear antibody.

a Two patients received an alprostadil infusion of 60 μg/day for 5 days within the 12 months preceding the blood sample collection.

According to LeRoy et al. (23), 12 (57.14%) and 9 (42.85%) patients presented with the dcSSc and the lcSSc subset, respectively. Other features of cutaneous involvement included Raynaud’s phenomenon, which was presented by the majority of patients (*n*=17, 80.95%), puffy fingers (*n*=12, 57.14%), digital tip ulcers and/or fingertip pitting scars (*n*=8, 38.09%), and calcinosis cutis (*n*=2, 9.52%). Gastrointestinal involvement was shown in 12 (57.14%) patients, presenting with dysphagia in 11 (52.38%) and constipation in 9 (42.85%) patients. Only one patient presented with chronic pericarditis, and another patient had a history of myocardial involvement with conduction abnormalities. The patients’ pack-year exposures are displayed in **Table 1**.

According to the ERS/ATS guidelines (30–32), 7 (33.33%) patients showed a restrictive pattern on spirometry and 8 (38.09%) patients a reduction in DLCO. Among the patients with restrictive patterns on spirometry and altered DLCO, seven and six patients, respectively, also displayed ILD on HRTC. The treatment strategies in our cohort are summarized in **Table 1**.

### 3.2 Serum concentrations of uPA and PAI-1 in SSc patients

The contribution of the uPA system to the pathogenesis of SSc is supported by several observations (6–10, 33). It is conceivable that serum modifications in the uPA system components could provide utility in guiding and monitoring the progression of SSc.

**Figure 1:**
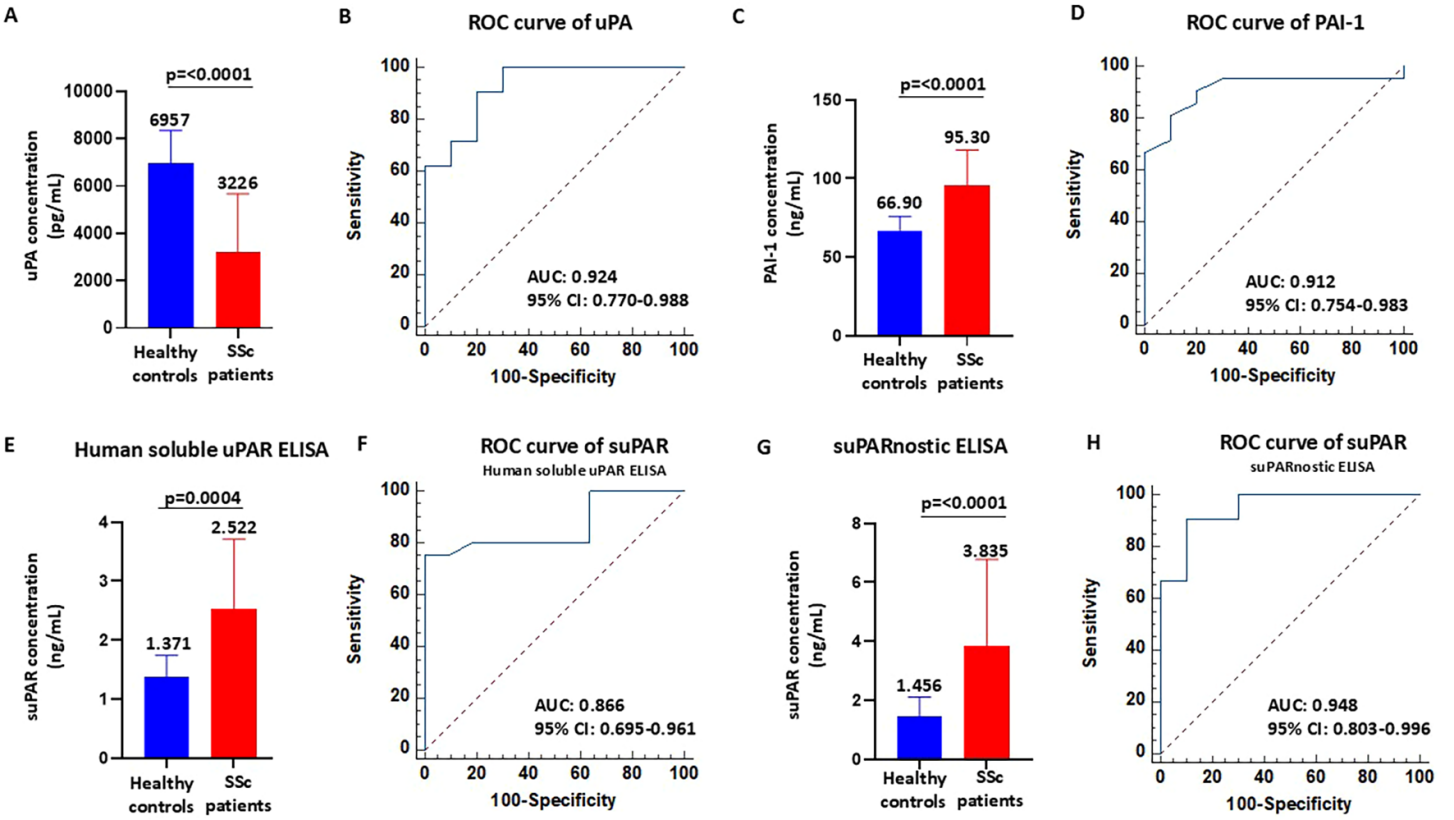
Serum concentrations of urokinase plasminogen activator (uPA), plasminogen activator inhibitor-1 (PAI-1), and soluble urokinase plasminogen activator receptor (suPAR) in patients with systemic sclerosis (SSc). **(A–D)** Serum levels of uPA **(A)** and PAI-1 **(C)** and receiver operating characteristic (ROC) analyses **(B, D)** in healthy controls and SSc patients. **(E, F)** Serum levels of suPAR measured with the Human suPAR immunoassay **(E)** and ROC analysis **(F)** in healthy controls and SSc patients. **(G, H)** Serum levels of suPAR measured with the suPARnostic assay **(G)** and ROC analysis **(H)** in healthy controls and SSc patients.

Firstly, the serum levels of uPA and PAI-1 were measured in healthy controls (*n*=10) and in patients with SSc (*n*=21). Of note is that uPA can exist in both the bound- and the unbound-receptor form in biological fluids. Here, we used the uPA ELISA kit in detecting the unbound form of uPA, also known as “free uPA.” We measured free uPA rather than the uPA–PAI-1 complex as free uPA represents the active form capable of binding uPAR and mediating pericellular proteolysis. In contrast, uPAR-bound uPA reflects the inhibited fraction of the enzyme. Assessing free uPA provides insights into the biologically active component of the fibrinolytic system, which may be particularly relevant to the vascular dysfunction and tissue remodeling process characteristics in SSc. As shown in **Figure 1A**, patients with SSc had significantly lower uPA concentrations (mean = 3,226 ± 2,444 pg/ml, median = 3,178 pg/ml, range = 6,590 pg/ml) than the healthy controls (mean = 6,957 ± 1,390 pg/ml, median = 6,986 pg/ml, range = 4,614 pg/ml). The ROC curve for serum uPA is shown in **Figure 1B**. The area under the curve (AUC) for uPA was 0.924 (95% CI = 0.770–0.988, *p* < 0.0001). The cutoff value was 6,321 pg/ml, at the highest Youden’s index (sensitivity + specificity − 1 = 0.7048), with a sensitivity of 90.48% and a specificity of 80.00%. For the serum PAI-1 levels, total PAI-1, comprising the active and latent forms, was measured. Total PAI-1 better reflects the overall inhibitory capacity of the fibrinolytic system. Although active PAI-1 may provide a more dynamic assessment, its levels can fluctuate more rapidly. Therefore, total PAI-1 is more stable and is widely used in clinical and translational studies investigating vascular and fibrotic diseases. As shown in **Figure 1C**, patients with SSc had significantly higher PAI-1 concentrations (mean = 95.30 ± 22.80 ng/ml, median = 103 ng/ml, range = 107 ng/ml) than the healthy controls (mean = 66.90 ± 9.049 ng/ml, median = 67 ng/ml, range = 31 ng/ml). The ROC curve for serum PAI-1 is shown in **Figure 1D**. The AUC for PAI-1 was 0.912 (95% CI = 0.754–0.983, *p* < 0.0001). The cutoff value was 75 ng/ml, at the highest Youden’s index (sensitivity + specificity − 1 = 0.7095), with a sensitivity of 80.95% and a specificity of 90.00%.

While these ROC curves suggest a strong discriminative potential of uPA, the small sample size warrants caution in the interpretation of these findings, which will require validation in larger, independent cohorts.

### 3.3 Serum concentration of suPAR in SSc patients and methodological impact

Although suPAR might be a valuable diagnostic and prognostic marker of SSc, only a few studies have assessed the serum suPAR levels and determined their clinical correlation with SSc complications (15–17). In addition, contrasting data obtained in previous studies are due to relevant differences between commercially available detection tests, such as Human suPAR ELISA and suPARnostic ELISA. The discrepancy between assays may be due to the varied ability to detect the various forms of suPAR (34, 35). In fact, circulating suPAR exists in different forms due to proteolytic cleavage and the interaction status with uPA: full-length suPAR (DI–DII–DIII), uPA-bound full-length suPAR (uPA/DI–DII–DIII), and the DII–DIII fragment. Firstly, we measured the serum concentrations of suPAR in healthy controls (*n*=10) and in patients with SSc (*n*=21) using Human suPAR ELISA. This assay uses a monoclonal capture antibody and polyclonal detection antibodies. As shown in **Figure 1E**, patients with SSc had significantly higher suPAR concentrations (mean = 2.522 ± 1.186 ng/ml, median = 2.356 ng/ml, range = 5.198 ng/ml) than the healthy controls (mean = 1.371 ± 0.371 ng/ml, median = 1.521 ng/ml, range = 1.087 ng/ml). The ROC curve for suPAR is shown in **Figure 1F**. The AUC for suPAR was 0.866 (95% CI = 0.770–0.988, *p* < 0.0001). The cutoff value was 1.824 ng/ml, at the highest Youden’s index (sensitivity + specificity − 1 = 0.7500), with a sensitivity of 75.00% and a specificity of 100.00%. Subsequently, we measured the serum concentrations of suPAR in healthy controls (*n*=10) and in patients with SSc (*n*=21) using suPARnostic ELISA that is based on a simplified double monoclonal antibody sandwich ELISA. As shown in **Figure 1G**, patients with SSc had significantly higher suPAR concentrations (mean = 3.835 ± 2.944 ng/ml, median = 3.072 ng/ml, range = 14.15 ng/ml) than the healthy controls (mean = 1.456 ± 0.652 ng/ml, median = 1.120 ng/ml, range = 1.685 ng/ml). The ROC curve for suPAR is shown in **Figure 1H**. The AUC for suPAR was 0.948 (95% CI = 0.803–0.996, *p* < 0.0001). The cutoff value was 2.260 ng/ml, at the highest Youden’s index (sensitivity + specificity − 1 = 0.8048), with a sensitivity of 90.48% and a specificity of 90.00%. These data indicate that both Human suPAR ELISA and suPARnostic ELISA can differentiate patients with SSc from healthy controls, albeit with significant differences in the quantitative determination and cutoff values. An illustration of the suPAR values obtained with the two assays for each patient is shown in **Supplementary Figure S1**. In 13 patients with SSc, the suPAR levels measured with suPARnostic ELISA were higher than those with Human suPAR ELISA, whereas only two SSc patients exhibited higher suPAR levels measured with Human suPAR ELISA compared with suPARnostic ELISA. In contrast, the suPAR levels measured with Human suPAR ELISA and suPARnostic ELISA overlapped in six patients with SSc.

### 3.4 Circulating TGF-β1 and VEGF in SSc patients and their association with the uPA system

**Figure 2:**
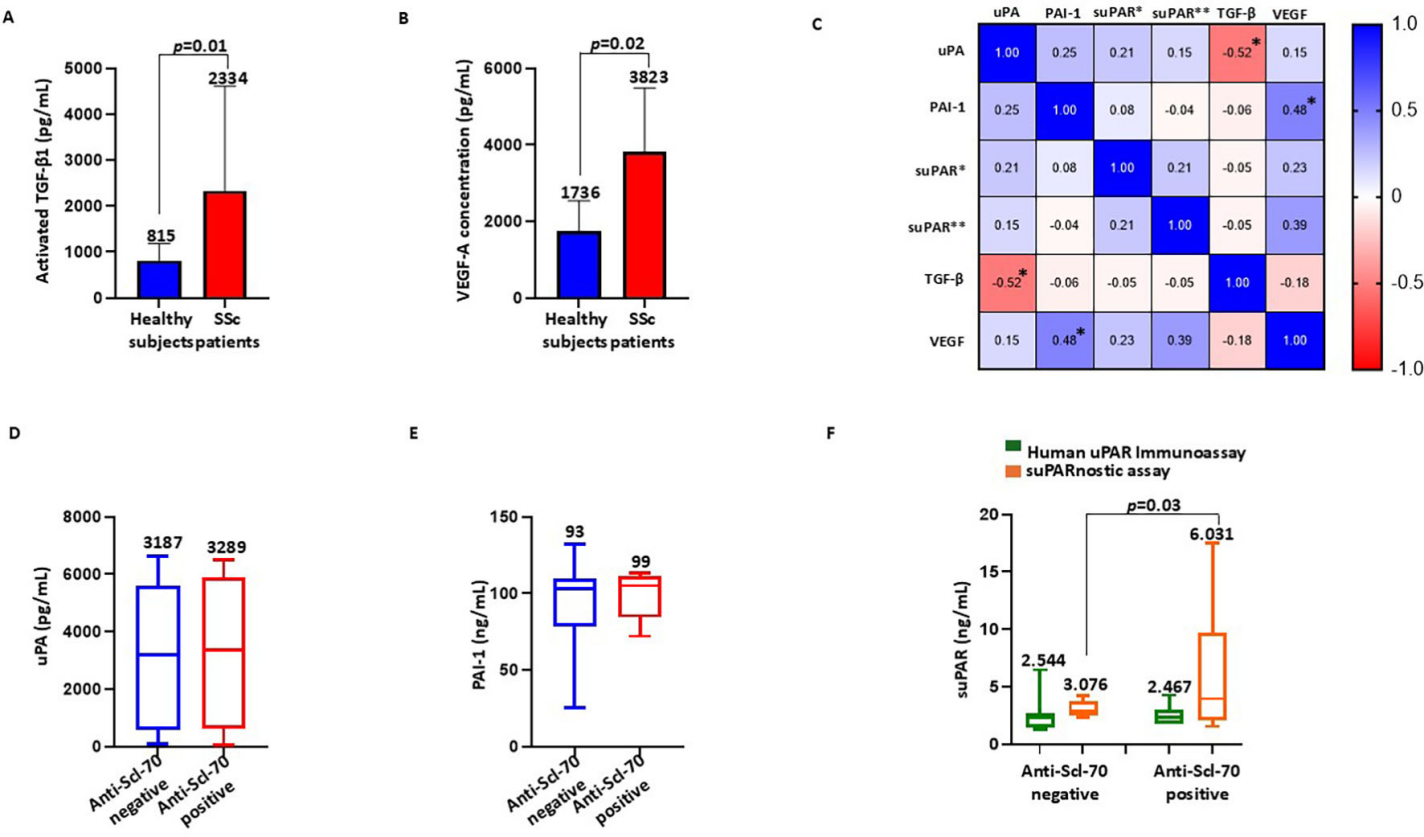
Association of the serum components of the urokinase plasminogen activator (uPA) system with the fibrotic markers and anti-Scl-70 antibodies. **(A, B)** Activated TGF-β1 **(A)** and VEGF-A **(B)** serum concentrations in healthy controls and systemic sclerosis (SSc) patients. **(C)** Correlation heatmap between circulating transforming growth factor beta 1 (TGF-β1), vascular endothelial growth factor (VEGF), uPA, plasminogen activator inhibitor-1 (PAI-1), and soluble urokinase plasminogen activator receptor (suPAR) measured with the Human suPAR immunoassay (suPAR*) and the suPARnostic assay (suPAR**). An *asterisk* indicates a statistically significant result, which is typically defined as an adjusted *p*-value ≤0.05. **(D)** Serum levels of uPA in anti-Scl-70-negative and anti-Scl-70-positive SSc patients. **(E)** Serum levels of PAI-1 in anti-Scl-70-negative and anti-Scl-70-positive SSc patients. **(F)** Serum levels of suPAR measured with the Human suPAR immunoassay and the suPARnostic assay in anti-Scl-70-negative and anti-Scl-70-positive SSc patients.

TGF-β is the central player in the pathogenesis of SSc through the regulation of fibrosis, inflammation, and vasculopathy (36). We estimated the concentrations of activated TGF-β1 in healthy controls (*n*=5) and in patients with SSc (*n*=21) with the ELISA methods. As shown in **Figure 2A**, patients with SSc had significantly higher concentrations of activated TGF-β1 (mean = 2,334 ± 2,277 pg/ml, median = 1,883 pg/ml, range = 11,169 pg/ml) than the healthy controls (mean = 815 ± 375 pg/ml, median = 655 pg/ml, range = 870 pg/ml). In parallel, we assessed the serum concentrations of VEGF in healthy controls (*n*=5) and in patients with SSc (*n*=21) using ELISA. As reported in **Figure 2B**, the serum VEGF levels significantly increased in patients with SSc (mean = 3,823 ± 1,664 pg/ml, median = 3,952 pg/ml, range = 5,980 pg/ml) compared with the healthy controls (mean = 1,736 ± 801 pg/ml, median = 1,599 pg/ml, range = 1,696 pg/ml). These results are in line with results from previous studies in which significant elevations in the plasma or serum VEGF levels in patients with SSc have been observed (37). Subsequently, we examined the correlation between circulating TGF-β1 and VEGF and the serum components of the uPA system by calculation of Spearman’s correlation and construction of a heatmap (**Figure 2C**). A negative correlation between uPA and activated TGF-β1 (Spearman’s *𝜌* = −0.52, *p*=0.008, BH-adjusted *p*=0.04) was found in the sera of patients with SSc, whereas a positive correlation was observed between PAI-1 and VEGF (Spearman’s *𝜌* = 0.48, *p*=0.01, BH-adjusted *p*=0.05). suPAR, measured using suPARnostic ELISA, showed a moderate correlation with VEGF (Spearman’s *𝜌* = 0.39, *p*=0.03, BH-adjusted *p*=0.15), which was not significant after correction for multiple comparisons.

### 3.5 Association of the serum components of the uPA system with anti-Scl-70 antibodies

Anti-Scl-70 antibodies are specific for SSc and play important diagnostic and prognostic roles in this disease (38). To evaluate the relationship between anti-Scl-70 antibodies and the uPA system, we divided the patient cohort into the anti-Scl-70-negative and anti-Scl-70-positive subgroups and analyzed the serum levels of uPA, PAI-1, and suPAR. As shown in **Figure 2D**, no significant difference was detected in the serum uPA between the anti-Scl-70-negative (mean = 3,187 ± 2,497 pg/ml, median = 3,187 pg/ml, range = 6,546 pg/ml) and anti-Scl-70-positive patients (mean = 3,289 ± 2,524 pg/ml, median = 3,368 pg/ml, range = 6,447 pg/ml). Similarly, there was no significant difference in the serum PAI-1 concentrations between the anti-Scl-70-negative (mean = 93.08 ± 26.72 ng/ml, median = 103 ng/ml, range = 107 ng/ml) and anti-Scl-70-positive patients (mean = 99.00 ± 15.37 ng/ml, median = 105 ng/ml, range = 41 ng/ml), as displayed in **Figure 2E**. **Figure 2F** presents the data on the serum levels of suPAR measured using both Human suPAR ELISA and the suPARnostic assay. No significant difference was detected in the serum suPAR measured using Human suPAR ELISA between the anti-Scl-70-negative (mean = 2.544 ± 1.298 ng/ml, median = 2.356 ng/ml, range = 5.198 ng/ml) and anti-Scl-70-positive patients (mean = 2.467 ± 0.947 ng/ml, median = 2.285 ng/ml, range = 2.541 ng/ml), whereas the serum suPAR levels measured with the suPARnostic assay were significantly increased in the anti-Scl-70-positive patients (mean = 6.031 ± 5.937 ng/ml, median = 3.939 ng/ml, range = 15.95 ng/ml) compared with the anti-Scl-70-negative patients (mean = 3.076 ± 0.626 ng/ml, median = 2.871 ng/ml, range = 1.865 ng/ml).

### 3.6 Association of the serum components of the uPA system with the scleroderma-type capillaroscopic pattern

**Figure 3:**
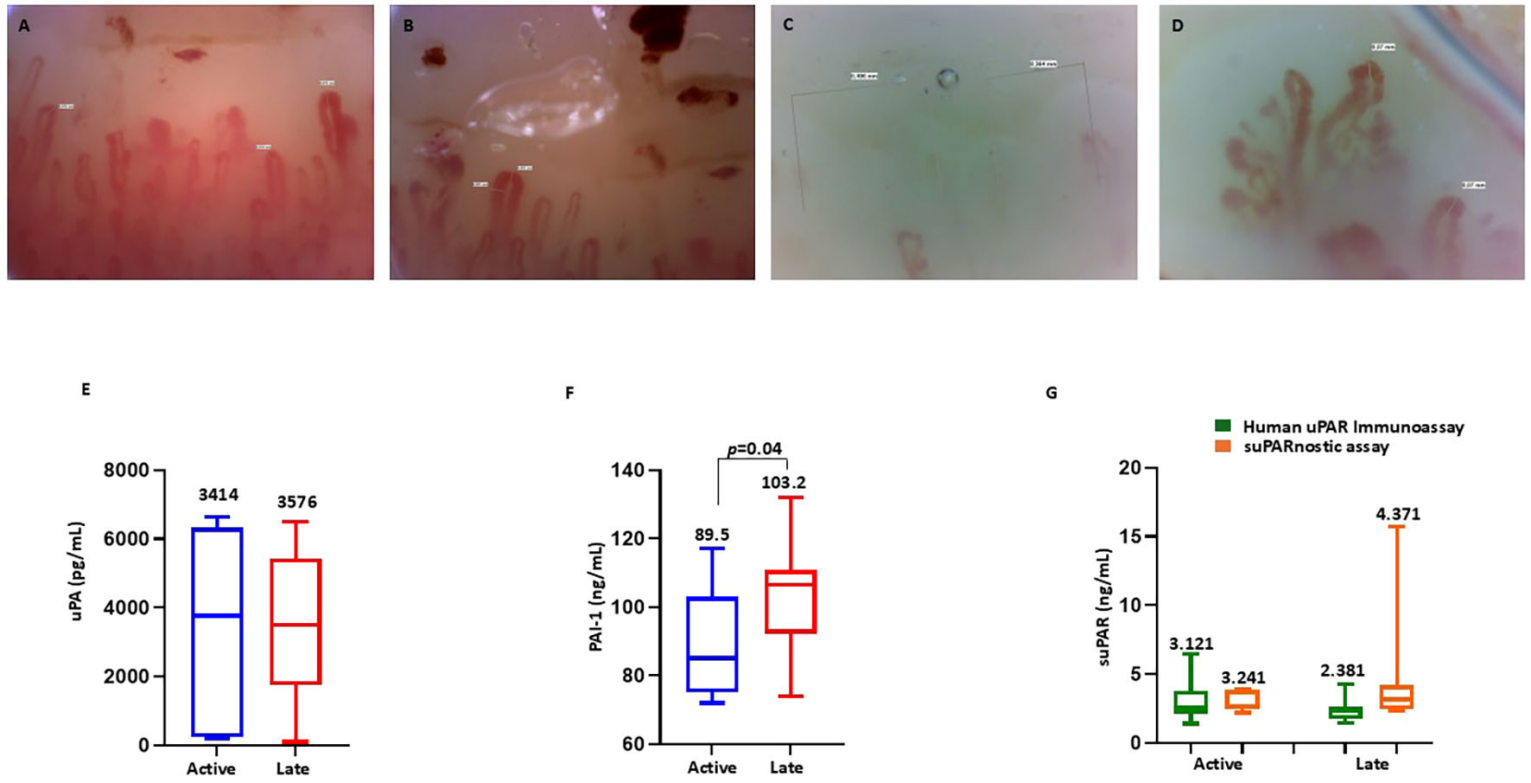
Nailfold videocapillaroscopy in patients with systemic sclerosis (SSc). **(A, B)** Active scleroderma pattern showing lowered capillary density, frequent giant capillaries (diameter > 50 mm), and microhemorrhages. **(C)** Late scleroderma pattern with further reduced capillary density and the absence of giant capillaries and microhemorrhages. **(D)** Abnormal capillary shape showing ramifications (neoangiogenesis). **(E)** Serum levels of urokinase plasminogen activator (uPA) in SSc patients with the active and late patterns. **(F)** Serum levels of plasminogen activator inhibitor-1 (PAI-1) in SSc patients with the active and late patterns. **(G)** Serum levels of soluble urokinase plasminogen activator receptor (suPAR) measured using the human suPAR immunoassay and the suPARnostic assay in SSc patients with the active and late patterns.

Vascular disease is crucial in patients with SSc; however, there is wide heterogeneity in its manifestations. At present, data on biomarkers able to assess vascular disease activity are limited (39). Hence, we performed NVC on the SSc cohort to evaluate a possible relationship between the capillaroscopic pattern and the serum components of the uPA system, thus assessing their ability to provide detailed insights into microvascular function. Overall, the SSc-early pattern was found in two patients, the SSc-active pattern was found in seven patients (**Figures 3A, B**), and the SSc-late pattern was found in 12 patients (**Figures 3C, D**). Given their small number, patients with the SSc-early pattern were excluded from this analysis. We evaluated the serum levels of uPA, PAI-1, and suPAR stratifying patients with SSc according to their capillaroscopic pattern. As shown in **Figure 3E**, no significant difference was detected in the serum uPA between patients with the SSc-active pattern (mean = 3,414 ± 2,895 pg/ml, median = 3,763 pg/ml, range = 6,437 pg/ml) and those with the SSc-late pattern (mean = 3,576 ± 2,173 pg/ml, median = 3,512 pg/ml, range = 6,403 pg/ml). In contrast, analysis of PAI-1 (reported in **Figure 3F**) showed that, in patients with the SSc-late pattern (mean = 103.2 ± 15.47 ng/ml, median = 106.5 ng/ml, range = 58.00 ng/ml), the serum levels were significantly higher than in those with the SSc-active pattern (mean = 89.57 ± 16.16 ng/ml, median = 85.00 ng/ml, range = 45.00 ng/ml). No significant difference was observed in the serum suPAR measured using Human suPAR ELISA between patients with the SSc-active (mean = 3.121 ± 1.666 ng/ml, median = 2.517 ng/ml, range = 5.072 ng/ml) and the SSc-late pattern (mean = 2.381 ± 0.714 ng/ml, median = 2.378 ng/ml, range = 2.823 ng/ml), as displayed in **Figure 3G**. There were higher suPAR levels analyzed with suPARnostic ELISA in patients with the SSc-late pattern (mean = 4.371 ± 3.834 = ng/ml, median = 3.113 ng/ml, range = 14.15 ng/ml) than in those with the SSc-active pattern (mean = 3.241 ± 0.722 ng/ml, median = 3.690 ng/ml, range = 1.677 ng/ml), although the difference was not statistically significant (**Figure 3G**).

### 3.7 Association of the serum components of the uPA system with microvascular abnormalities

We compared the capillaroscopic variables and the serum components of the uPA system using Spearman’s rank correlation. **Table 2** summarizes the correlation coefficients and their significance between the serum uPA, PAI-1, and suPAR levels and the capillaroscopic indicators of microvascular impairment. A negative correlation was observed between uPA and interstitial edema (Spearman’s *𝜌* = −0.471, *p*=0.010, BH-adjusted *p*=0.04), which remained significant after adjustment. PAI-1 showed a positive correlation with interstitial edema (Spearman’s *𝜌* = 0.361, *p*=0.050, BH-adjusted *p*=0.100) and a negative correlation with loop length (Spearman’s *𝜌* = −0.645, *p*=0.001, BH-adjusted *p*=0.004). The association between PAI-1 and loop length remained significant after correction, whereas the correlation with interstitial edema lost statistical significance following BH adjustment. Furthermore, suPAR, measured with the suPARnostic assay, was positively correlated with apical diameter (Spearman’s *𝜌* = 0.472, *p*=0.021, BH-adjusted *p*=0.084), an important indicator of structural capillary alterations in patients with SSc. Although the unadjusted *p*-value suggested significance, this correlation did not remain significant after multiple testing corrections.

**Table 2:** Correlation between the capillaroscopic parameters and the serum concentrations of urokinase plasminogen activator (uPA), plasminogen activator inhibitor-1 (PAI-1), and soluble urokinase plasminogen activator receptor (suPAR).

	uPA		PAI-1		Human uPAR immunoassay		suPARnostic assay	
	*r*	*p* (*p*_adj_)	*r*	*p* (*p*_adj_)	*r*	*p* (*p*_adj_)	*r*	*p* (*p*_adj_)
Intercapillary distance	−0.077	0.377 (0.460)	0.185	0.224 (0.460)	0.025	0.460 (0.460)	−0.025	0.460 (0.460)
Apical diameter	−0.104	0.335 (0.335)	−0.237	0.164 (0.328)	0.151	0.269 (0.335)	0.472	0.021 (0.084)
Loop length	−0.110	0.327 (0.406)	−0.645	0.001 (0.004)	−0.167	0.247 (0.406)	0.059	0.406 (0.406)
Internal diameter	0.098	0.345 (0.49)	−0.321	0.090 (0.36)	0.023	0.463 (0.49)	0.006	0.490 (0.49)
External diameter	0.089	0.358 (0.466)	−0.277	0.126 (0.466)	−0.021	0.466 (0.466)	0.046	0.426 (0.466)
Capillary density	0.038	0.436 (0.477)	−0.190	0.211 (0.477)	−0.149	0.264 (0.477)	−0.014	0.477 (0.477)
Capillary distribution	−0.154	0.258 (0.497)	0.169	0.238 (0.497)	0.050	0.417 (0.497)	−0.002	0.497 (0.497)
Capillary morphology	0.084	0.362 (0.362)	0.228	0.166 (0.362)	−0.092	0.350 (0.362)	0.124	0.301 (0.362)
Hemorrhages	0.158	0.253 (0.287)	−0.340	0.071 (0.174)	0.316	0.087 (0.174)	0.134	0.287 (0.287)
Interstitial edema	−0.471	0.010 (0.04)	0.361	0.050 (0.100)	−0.030	0.450 (0.450)	0.230	0.164 (0.218)

Correlation was assessed using Spearman’s correlation analysis and reported as the correlation coefficient (*r*). A *p*-value ≤0.05 was considered significant. Statistical significance was assessed using adjusted *p*-values (*p*_adj_).

### 3.8 Association of the serum components of the uPA system with pulmonary complications

To determine whether the components of the uPA system could be used as biomarkers for severe pulmonary involvement in SSc, we investigated the association between the serum uPA, PAI-1, and suPAR levels and the presence of lung interstitiopathy, restrictive lung disease, and reduced DLCO. Among all the serum components of the uPA system, suPAR, measured using the suPARnostic assay, showed the most notable association (**Table 3**). In agreement with previous reports (16), the suPAR values were positively associated with reduced DLCO (Spearman’s *𝜌* = 0.404, *p*=0.050, BH-adjusted *p*=0.2). However, this association did not remain statistically significant after correction for multiple comparisons, suggesting that the observed relationship should be interpreted with caution.

**Table 3:** Correlation between pulmonary complications and the serum concentrations of urokinase plasminogen activator (uPA), plasminogen activator inhibitor-1 (PAI-1), and soluble urokinase plasminogen activator receptor (suPAR).

	uPA		PAI-1		Human uPAR immunoassay		suPARnostic assay	
	*r*	*p* (*p*_adj_)	*r*	*p* (*p*_adj_)	*r*	*p* (*p*_adj_)	*r*	*p* (*p*_adj_)
Lung interstitiopathy	−0.076	0.386 (0.498)	−0.394	0.059 (0.236)	−0.153	0.279 (0.498)	−0.001	0.498 (0.498)
Restrictive lung disease	−0.060	0.410 (0.487)	−0.323	0.103 (0.342)	0.246	0.171 (0.342)	−0.008	0.487 (0.487)
Reduced DLCO	−0.060	0.409 (0.409)	−0.231	0.186 (0.248)	−0.268	0.150 (0.248)	0.404	0.050 (0.2)

Correlation was assessed using Spearman’s correlation analysis and reported as the correlation coefficient (*r*). A *p*-value ≤0.05 was considered significant. Statistical significance was assessed using adjusted *p*-values (*p*_adj_).

*DLCO*, diffusing capacity of the lung for carbon monoxide.

## 4 Discussion

The aim of the present study was to investigate the serum concentrations of the uPA system components and assess potential associations with the clinicopathological and prognostic parameters in patients with SSc.

**Figure 4:**
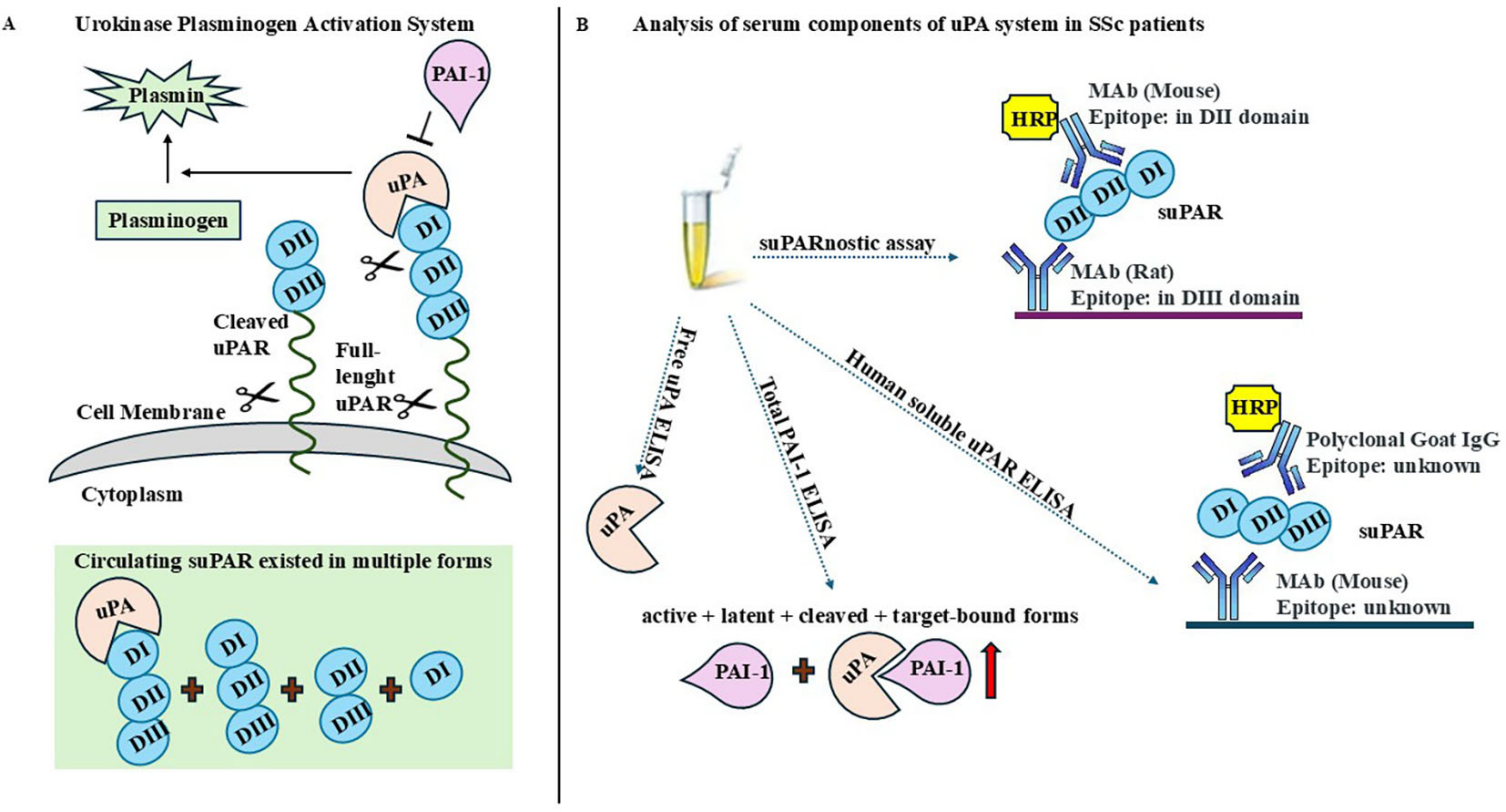
Overview of the urokinase plasminogen activator (uPA) system and detectable molecular forms. **(A)** Schematic representation of the organization of the uPA system and the principal circulating isoforms of soluble urokinase plasminogen activator receptor (suPAR). **(B)** Specific molecular forms of uPA, plasminogen activator inhibitor-1 (PAI-1), and suPAR detectable with the ELISA kits employed in this study.

We found that a low level of circulating uPA and high levels of PAI-1 and suPAR could serve as potential biomarkers to differentiate patients with SSc from healthy controls in clinical practice. To aid the interpretation of our results, we developed a figure that summarizes the key findings on the organization of the uPA system, the multiple circulating forms of suPAR, and the specific molecular forms of uPA, PAI-1, and suPAR detectable with the ELISA kits used in this study (see **Figure 4**).

In this study, the choice to measure free uPA and total PAI-1 has relevant pathophysiological implications in the context of SSc. Free uPA reflects the active form involved in pericellular proteolysis and ECM turnover, while total PAI-1 represents the overall inhibitory capacity of the system. The reduced free uPA and increased total PAI-1 levels observed in our cohort suggest a shift toward an anti-fibrinolytic state, potentially contributing to persistent fibrosis and vascular dysfunction in SSc. Two ELISA tests were used for the detection of suPAR: Human suPAR ELISA and the suPARnostic assay. Although both ELISA kits were able to differentiate patients with SSc from healthy controls, the tests were not superimposable. In fact, the analysis performed using the suPARnostic assay showed higher suPAR levels and better risk of discrimination than Human suPAR ELISA, as already demonstrated in previous studies (34, 35). Human suPAR ELISA uses a monoclonal capture antibody and polyclonal detection antibodies; however, the specific suPAR forms detected by this assay are unknown. The suPARnostic assay consists of a monoclonal capture antibody targeting the DIII subunit and a monoclonal detection antibody targeting the DII subunit, thus capturing both the DI–DII–DIII and DII–DIII fragments, but not uPA/DI–DII–DIII. The discrepancy between these methods could be explained by their differing abilities to detect various forms of suPAR.

In the patients with SSc analyzed in the study, the serum TGF-β1 and VEGF levels were elevated and were significantly associated with the elements of the uPA system. The observed negative correlation between uPA and TGF-β1 further supports the notion that uPA exerts anti-fibrotic effects. Through the conversion of plasminogen to plasmin, uPA promotes the degradation of fibrin and the ECM components, thereby limiting excessive matrix deposition. These findings are consistent with previous *in vivo* data demonstrating that uPA overexpression attenuates fibrotic progression, underscoring its potential protective role in SSc (40). For these reasons, decreased levels of circulating uPA could indicate a fibrotic disease. The consumption or the reduction of free circulating uPA could be caused by the higher suPAR levels that act as a “decoy” receptor for uPA, thus impairing the function of the uPA system in endothelial cells and leading to microvascular abnormalities and impaired fibrosis (15).

Serum PAI-1 was positively correlated with serum VEGF, but not with serum TGF-β1. This finding likely reflects the dysfunctional angiogenic response characteristics of SSc, in which elevated VEGF fails to promote effective neovascularization due to endothelial damage and the concomitant increase in PAI-1, an anti-angiogenic mediator (41). Given that TGF-β1 promotes the expression of PAI-1 and that the pro-fibrotic effects of TGF-β are partly mediated by PAI-1 (42), it is paradoxical that no significant correlation was observed between the serum TGF-β1 and PAI-1 levels. This discrepancy may reflect the predominantly local and context-dependent actions of TGF-β1. TGF-β1 exerts its biological effects mainly through autocrine and paracrine signaling within specific tissues. Moreover, the expression of PAI-1 is regulated by multiple stimuli beyond TGF-β1, including inflammatory cytokines, metabolic factors, and hypoxic stress, all of which can independently influence systemic PAI-1 concentrations. Differences in the cellular sources also contribute to this divergence, as serum TGF-β1 is derived largely from platelets and immune cells, whereas circulating PAI-1 originates mainly from endothelial cells and adipose tissue (43). Thus, the lack of correlation between serum TGF-β1 and PAI-1 likely reflects a compartmentalized regulation and the intricate interplay between the systemic and tissue-specific mechanisms involved in fibrotic signaling. Alternatively, the absence of a correlation may be due to the limited cohort size.

suPAR, measured using the suPARnostic assay, was found to be positively correlated with the serum VEGF levels, and although a weak correlation, this confirms the direct relationship between uPAR and angiogenesis. In particular, VEGF signaling is funneled through uPAR to control proteolysis and the integrin-dependent cell migration during the angiogenic response (44).

Further correlations with the laboratory and clinical parameters showed that the suPARnostic assay outperformed the Human suPAR ELISA. This was based on the higher suPAR levels in the anti-Scl-70-positive patients when compared with the Human suPAR ELISA. These results emphasized the role of suPAR detected by the suPARnostic assay as a potential marker of disease severity. As anti-Scl70 antibodies occur before manifestation of the clinical signs of the disease, it is conceivable that suPAR is also a potential early diagnostic marker.

In nailfold capillaroscopy, high levels of suPAR measured using the suPARnostic assay were observed in patients with the late pattern, which suggests a more advanced stage of microvascular damage. We also observed a significant increase in the PAI-1 levels in patients with the late pattern compared with those with the early pattern. These data are in agreement with previous reports demonstrating that the PAI-1 levels were significantly increased in diabetic patients with microvascular injury (45).

In addition, a thorough capillaroscopy examination showed that the levels of suPAR measured with the suPARnostic assay presented a moderate positive correlation with the apical diameter values. The apical diameter refers to the width of a capillary at its tip and is part of the analysis of capillary morphology, which aids in the diagnosis and monitoring of SSc-related microvascular abnormalities.

A recent study found that a high concentration of suPAR correlated with a severe reduction in DLCO (16). Corroborating these findings, the present study showed that only the suPAR measured using the suPARnostic assay was positively correlated with the occurrence of a reduced DLCO.

The data presented in this study are reliable as the patients with SSc were clinically well characterized and blood sampling was performed under controlled conditions. Nevertheless, a number of limitations must be acknowledged. The serum levels of uPA, PAI-1, and suPAR were assessed in a relatively small cohort, which may have influenced the robustness of the conclusions. In addition, the modest sample size and the clinical heterogeneity of SSc may have limited the generalizability of our findings. Accordingly, these results should be considered exploratory and hypothesis-generating, requiring validation in larger, independent cohorts.

Another potential limiting factor in our study is ongoing treatment. Majority of the patients included in this study were receiving potent immunomodulatory or vasoactive agents, such as mycophenolate and bosentan, as well as corticosteroids. It was therefore not possible to determine whether the observed biomarker levels (uPA, PAI-1, and suPAR) reflect disease-related mechanisms or treatment effects. Corticosteroids can increase the PAI-1 levels, potentially reducing fibrinolytic activity, whereas mycophenolate may indirectly normalize fibrinolysis by reducing inflammation (46). Bosentan, by modulating the endothelial function, could also affect the fibrinolytic balance (47). These treatment-related influences should be considered when interpreting our findings.

In conclusion, our study demonstrates that the suPARnostic assay is a diagnostic method that enhanced the accuracy of the measurement of suPAR in SSc. Indeed, the serum suPAR concentrations, detected using this method, correlated with the disease severity and organ damage in patients with SSc. In addition, evaluation of the serum free uPA and PAI-1 levels could accompany the measurement of suPAR in clinical practice, probably through the development of a diagnostic algorithm.

Further studies could provide prognostic cutoff values for suPAR in chronic inflammation, as already done in acute inflammatory diseases (48). To ensure reliable analysis, it is necessary to develop new techniques capable of detecting the different circulating forms of suPAR.

## Data Availability

The raw data supporting the conclusions of this article will be made available by the authors, without undue reservation.
