# Supplementary material for: Serum profiling of uPA, PAI-1, and suPAR in systemic sclerosis: a preliminary study on analytical aspects and associations with microvascular and fibrotic manifestations
Source: Front Immunol. 2025 Dec 2;16:1697785. doi: 10.3389/fimmu.2025.1697785 (PMC12705569; doi:10.3389/fimmu.2025.1697785)
Supplement: Supplementary file 1 [file Table1.docx]

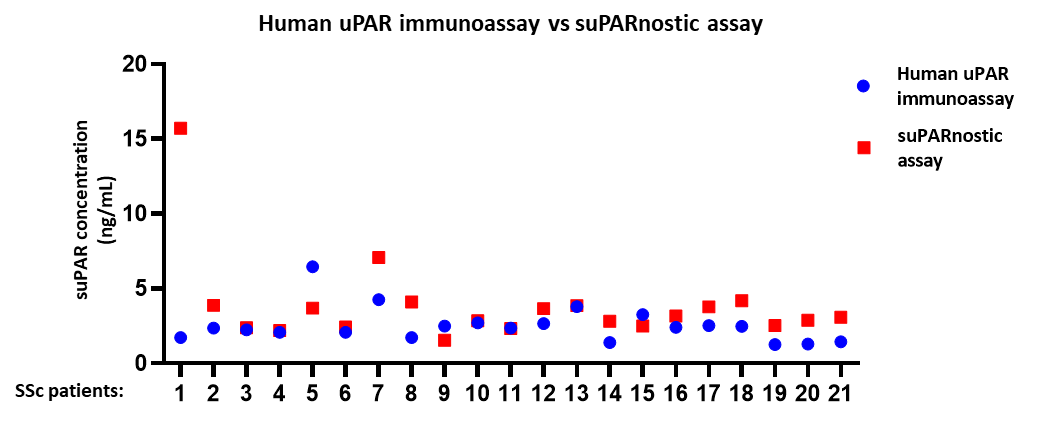


**Supplementary Figure S1.** An illustration of suPAR values obtained with the two assays in each SSc patient. In 13 patients, suPAR levels measured by suPARnostic ELISA were higher than Human soluble uPAR ELISA, whereas only 2 SSc patients exhibited higher suPAR levels measured by Human soluble uPAR ELISA as compared to suPARnostic ELISA. By contrast, suPAR levels measured by Human soluble uPAR ELISA and suPARnostic ELISA overlapped in only 6 SSc patient.
